# Supplementary material for: Targeted Nanoparticle Photodynamic Diagnosis and Therapy of Colorectal Cancer
Source: Int J Mol Sci. 2021 Sep 10;22(18):9779. doi: 10.3390/ijms22189779 (PMC8466279; doi:10.3390/ijms22189779)
Supplement: Supplementary file 1 [file ijms-22-09779-s001.zip › ijms-1363402-supplementary/Simelane Supplementary Material/Simelane Revision 2 Supplementary Material.pdf]

Supplementary Material: Targeted Nanoparticle Photodynamic Diagnosis and Therapy of Colorectal Cancer

Nokuphila Winifred Nompumelelo Simelane[1], Cherie Ann Kruger[1*] and Heidi Abrahamse[1]

[1]Laser Research Centre, Faculty of Health Sciences, University of Johannesburg, Doornfontein 2028, South Africa.

*Correspondence: cherier@uj.ac.za; Tel.: +27-11-559-6860.

1. MATERIALS & METHODS

1.1 Trypan Blue Exclusion Test

The trypan blue exclusion assay was utilised to stain dead cells to determine the number of cell viability. In this assay, Trypan blue stain is vital is excluded from viable cells that possess intact cell plasma membrane but can enter dead cells. In this assay, a 1:1 dilution of the cell suspension using a 0.4% Trypan Blue stain was used to perform trypan blue exclusion. The diluted cell suspension was loaded onto a disposable Countess® cell counting chamber slide and were evaluated with an Invitrogen Countess® II FL Automated Cell Counter to quantify the number of viable cells per ml.

1.2 LDH Cellular Cytotoxicity and Membrane Integrity Assay

In this study, to measure PDT-induced cytotoxicity, the presence of LDH released in the media was measured as an indicator for cell membrane damage and cytotoxicity, upon lysis. The CytoTox 96® Non-Radioactive Cytotoxicity Assay (Anatech: Promega, PRG1780) was employed to assess the cell membrane integrity. Briefly, after 24hrs post PDT treatment, this non-homogenous colorimetric assay was performed according to the manufacturer's instructions, where 50 µl of complete cell culture media supernatant from each experimental and control culture plate was removed and mixed with 50 µl of LDH Reconstituted Substrate Mix in a flat 96 well clear bottom plate. To determine cellular lysis and cytotoxicity, the LDH produced was detected by measuring LDH absorbance at 490 nm using a spectrophotometer (Perkin Elmer, Victor^3, 1420 Multilabel Counter).

1.3 Chemical Synthesis of the Final Multifunctional Tumor Targeted Bioactive Nanoconjugate (BNC)

Anti-GCC Ab (Abcam: ab122404) and AuNP-S-PEG5000-NH_2 (Sigma-Aldrich: 765309) which contained 2.85 X 10^{15} AuNPs per ml (Sigma-Aldrich 765309) were both conjugated to $ZnPcS_4$ using methods adapted from [1]. Briefly, working concentration of 0.0005 M $ZnPcS_4$ (Santa Cruz®: sc-264509A) (%w/v) in 0.001 M Phosphate Buffer Saline (PBS) was prepared and diluted as needed. 1 ml of AuNP-S-PEG5000-NH_2 (Sigma-Aldrich: 765309) which contained 2.85×10^{15} AuNPs per ml was added to 1 ml of 0.0005 M stock $ZnPcS_4$. It was vortexed at 1 500 rpm at room temperature overnight to promote spontaneous ligand exchange (between Au and PS tetra sulphides) and adsorption (disulphide bond between PEG and PS). It was purified by micro-centrifugation at 15 200 rpm for 1 hour. The supernatant was discarded and the pellet which contained the conjugated $ZnPcS_4$ and AuNP-S-PEG5000-NH_2 was re-suspended in 1 ml PBS.

200 µg/ml of Anti-GCC Ab (Abcam: ab122404) was activated using covalent mode carbodiimide crosslinker two-step coupling EDC and NHS chemistry. The activated c' terminus succinimidyl ester on the Anti-GCC Ab

reacted was then able to react with the amine group (NH_2) on the AuNPs, already bound in the $ZnPcS_4$ – AuNP-S-PEG5000-NH_2 conjugate, and so when mixed together, formed a stable amide bond [2]. This method ensured the correct orientation of the bio-targeting antibody, i.e.: the c' terminus was bound to the amine functionalized AuNPs, while the n' terminus antigenic sites remained free and functional for active targeting. The final BNC ($ZnPcS_4$ – AuNP-S-PEG5000-NH_2 – Anti-GCC Ab) was then subjected to various molecular characterization assays including UV-Visible and FT-IR Spectroscopy, DLS and ZP, as well as immunofluorescent staining subcellular localization and uptake confirmation assays, as described below.

1.4 Molecular Characterization of the Final Multifunctional Tumour Targeted Bioactive Nanoconjugate (BNC)

1.4.1 UV Visible Spectroscopy

To detect the interaction, as well as confirm the attachment of the Anti-GCC mAb and $ZnPcS_4$ PS to the surface of the AuNP-S-PEG5000-NH_2, the UV visible spectra of the final BNC and various controls ($ZnPcS_4$, AuNP-S-PEG5000-NH_2, Anti-GCC Ab, $ZnPcS_4$ – AuNP-S-PEG5000-NH_2) were obtained using a Jenway Genova Nano Plus Life Science Spectrophotometer. For this purpose, the absorption and fluorescent spectra were analysed by using spectrum/purity scan mode within the 198 to 800nm spectral region and 220nm protein direct UV option. The amount of mAb and $ZnPcS_4$ PS, as well as number of bound AuNPs in the final BNC was confirmed by comparing initial spectra of unbound chemical components ($ZnPcS_4$, AuNP-S-PEG5000-NH_2, Anti-GCC Ab) at known concentrations with final spectra obtained from the bound final BNC.

1.4.2 Fourier Transform Infrared (FTIR) Spectroscopy

AuNP-S-PEG5000-NH_2 – $ZnPcS_4$ conjugate underwent FTIR analysis using the Nicolet iS50 FT-IR Spectrometer (Thermo Scientific) to confirm the existence of strong Au-S via ligand exchange and absorption processes, when compared to AuNP-S-PEG5000-NH_2 alone. Furthermore, the final BNC also underwent FTIR analysis to confirm the formation of amide bonds in comparison to AuNP-S-PEG5000-NH_2 FTIR spectra alone. The infrared spectra results were read at 400 to 4000 cm^{-1} frequency range with 25 scans using far infra-red solution software.

1.4.3 Dynamic light scattering (DLS) and Zeta potential (ZP)

The particle size and zeta potential of the functionalized BNC and controls were performed in triplicate (with 15 runs each), with a Malvern Zetasizer Nano ZS (Malvern Instruments, Malvern UK), equipped with a 4mW He-Ne laser of 633 nm wavelength. Twelve microliters of each sample were loaded into a clean, scratch free plastic Zeta 3 x 3 mm dip cell cuvette, which had in-built electrodes capable of DLS and Zeta measurements. All analyzed samples were heterogeneous or homogenous 10 to 50 µg/ml diluted suspensions in PBS (diluted in deionized water). All experiments were performed at 25°C, at a 13° and 173° angle. Samples analyzed and compared consisted of AuNP-S-PEG5000-NH_2, $ZnPcS_4$, $ZnPcS_4$ – AuNP-S-PEG5000-NH_2, Anti-GCC mAb and BNC.

2. RESULTS

2.1 $ZnPcS_4$ PS and PDT Dose Response Assays

2.1.1 Cellular Viability Assessment: Trypan Blue Exclusion Test

The Trypan Blue Exclusion test is widely used to enumerate viable cells present in cell suspension. It relies on the principle of Trypan blue dye accumulation in nonviable cells do so, due to their damaged membranes, and viable cells do not take up the trypan blue dye, due to their intact cell membranes [3].

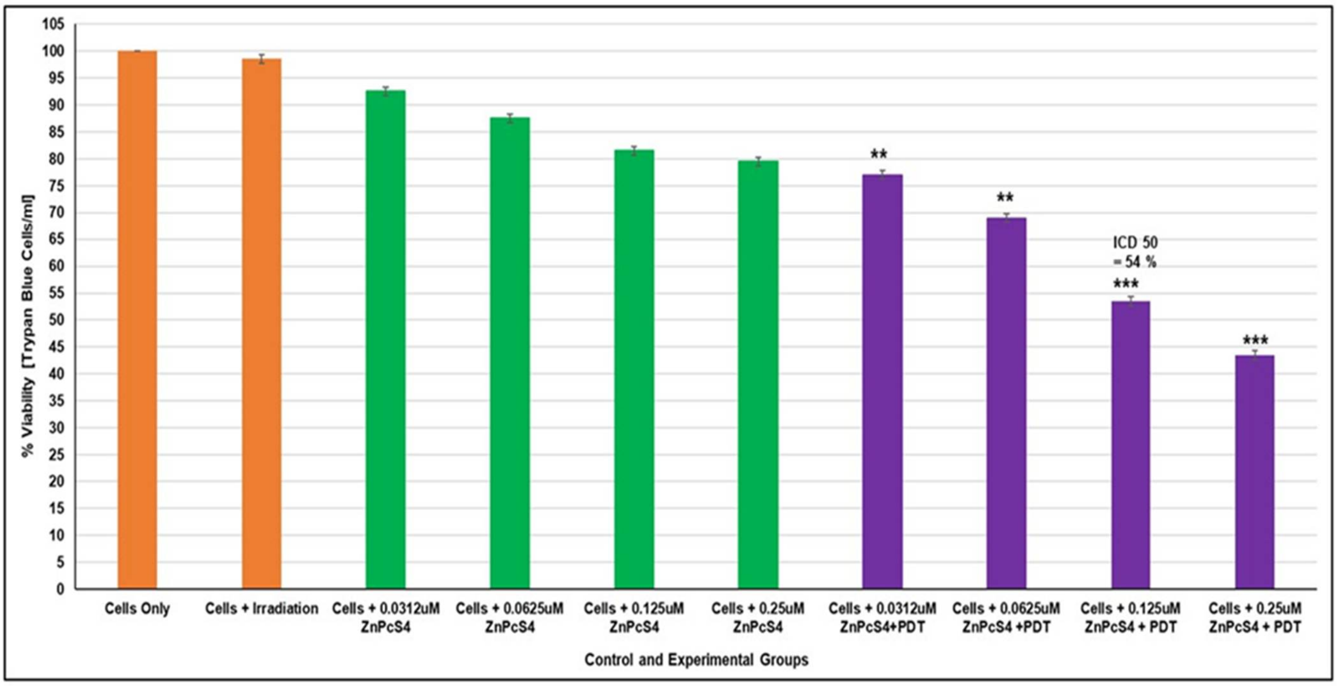

Figure S1: Trypan blue exclusion cell viability assay. Cell viability response of CaCo-2 cells after PDT treatment with $ZnPcS_4$ PS at varying concentrations, demonstrating a decrease in cell viability in a dose dependent manner.

To assess the effect of $ZnPcS_4$ PS PDT dose responses on the CRC cells viability after treatment, various controls and experimental groups were subjected to Trypan blue exclusion staining. As shown in Figure S1, for control CRC cells treated with different concentrations of $ZnPcS_4$ PS, without laser light irradiation, no significant dose dependent decreases in cell viability were observed, indicating that $ZnPcS_4$ in its inactivated form, did not exhibit obvious dark toxicity on CRC cells. Furthermore, control groups of CRC cells exposed to laser irradiation at 673 nm and fluency of 10 J/cm^2 in the absence of PS, noted no significant cytotoxic effects as an insignificant decrease in cell viability was observed when compared to the reference control group of cells only. This is consistent with literature, it was evidenced that laser irradiation at 673 nm with a fluency 10 J/cm^2, without any administration of a PS, does cause cellular damage to CRC cells [4]. In contrast, the PDT irradiated experimental CRC groups treated with varying concentrations of the $ZnPcS_4$ PS and 673 nm laser irradiation at 10 J/cm^2, showed a significant decreases in cellular viability which was dose-dependent (Figure S1). Within these PDT treated experimental groups, the most significant decrease in cell viability was evident in cells incubated with 0.25 µM $ZnPcS_4$ PS and laser irradiation at 10 J/cm^2 with enumeration results being 44%***, indicating that at this threshold concentration CRC PDT treatment could eradicate cells beyond cellular responses detection, due to the induction of severe cell death. Notably, at a concentration of 0.125 µM $ZnPcS_4$ PS, within the CRC PDT treated experimental groups, significant cellular damage occurred,

however, 54%** of the cells were found to be viable and so from this assay, it was the recommended ICD_{50} concentration of the $ZnPcS_4$ PS that did not adversely affect cell viability in order to measure final biological responses when conjugated in the BNC.

2.1.2 Cellular Cytoxicity Assessment: LDH Cellular Cytotoxicity and Membrane Integrity Assay

To evaluate the potential cytotoxicity of the $ZnPcS_4$ PS PDT on CRC various control and experimental groups were treated with different concentrations of $ZnPcS_4$ PS and 24h post irradiation; groups were subjected to Lactate Dehydrogenase - LDH membrane damage integrity analysis.

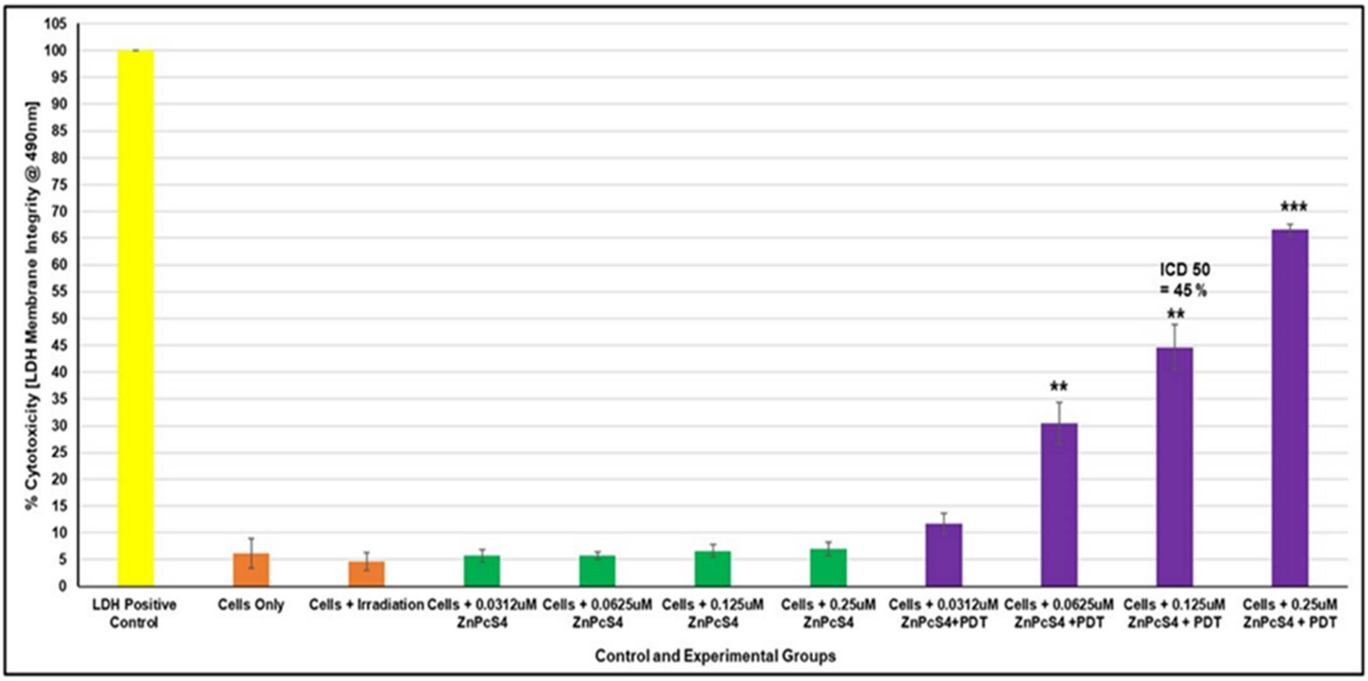

Figure S2: *In vitro* cytotoxicity effect of control and experimental groups of $ZnPcS_4$ PS drug at different concentrations with and without 673 nm laser irradiation at 10 J/cm^2, measured by LDH membrane integrity assay.

As shown in Figure S2, the control group of CRC cells treated with laser irradiation at 673 nm and fluency of 10 J/cm^2 only noted no significant increase in cellular cytotoxicity when compared to the LDH positive control group of cells only. These results were in agreement with studies performed by Manoto *et al.* (2012) that demonstrated no obvious cytotoxicity in CRC cells that were subjected to laser irradiation at 673 nm, with a fluency 10 J/cm^2, without any addition of a $ZnPcS_4$ PS [4] . Observation of the results of control groups treated with the various concentrations of $ZnPcS_4$ without laser irradiation, revealed a slight dose dependent cellular cytotoxicity increase, however it remained non-significant (Figure S2). Once again, this phenomenon showed that the $ZnPcS_4$ PS in its inactivated form when administered alone (without laser irradiation), within these particular concentration ranges, does not induce cytotoxic effects or exerts cellular damage within CRC cells. However, when 673nm laser irradiation at 10 J/cm^2 was used to irradiate PDT experimental groups which consisted of CRC cells treated at varying concentrations of the PS a significant increase in cellular cytotoxicity in a dose-dependent manner was observed (Figure S2). In particular, cells that received 0.25 µM $ZnPcS_4$ PS within PDT treated experimental groups demonstrated the most significant increase in cellular cytotoxicity at 67%***, suggesting that at this particular concentration, CRC PDT treatment could obliterate cells beyond cellular response detection, due to severe cytotoxicity induced. Within the PDT treated experimental groups,

CRC cells that received 0.125 µM $ZnPcS_4$ PS also noted significant cytotoxicity, however it was only 45%**, and so it was the recommended ICD_{50} concentration of the $ZnPcS_4$ PS that did not adversely affect cell viability in order to measure final biological responses when conjugated in the BNC.

2.2. Molecular Characterization of the Final Multifunctional Tumour Targeted Bioactive Nanoconjugate (BNC)

2.2.1. UV Visible Spectroscopy

Table S1: Absorbance fold fall comparison to determine the final bound Anti-GCC Ab protein concentration within the BNC ($ZnPcS_4$ - AuNP-PEG5000-SH-NH_2 - Anti-GCC Ab)

Sample	UV Absorbance at 280nm	Fold Fall	Concentration
Anti-GCC Ab	1.8255		200 µg/ml
AuNP	0.0666		
$ZnPcS_4$	0.2219		
$ZnPcS_4$ - AuNP - Anti-GCC Ab	0.5705		
$ZnPcS_4$ - AuNP - Anti-GCC Ab, calculation eliminating any protein presence from $ZnPcS_4$ and AuNP	0.5705 - 0.2219 - 0.0666 = 0.282	6.47	30.91 µg/ml

2.2.2 FT-IR Spectroscopy

FTIR spectroscopy is a widely used technique in structural identification, and was used for bond confirmatory analysis within the final BNC [5]. Spectral analysis of AuNP-S-PEG5000-NH_2 + $ZnPcS_4$ revealed a C-S (1050-1200 cm^{-1}) stretch shift, signifying that AuNP-S-PEG5000-NH_2 had lost their C-S groups to bond with the $ZnPcS_4$ PS, which represents Au-S ligand exchange bond (Figure S3) [6,7].

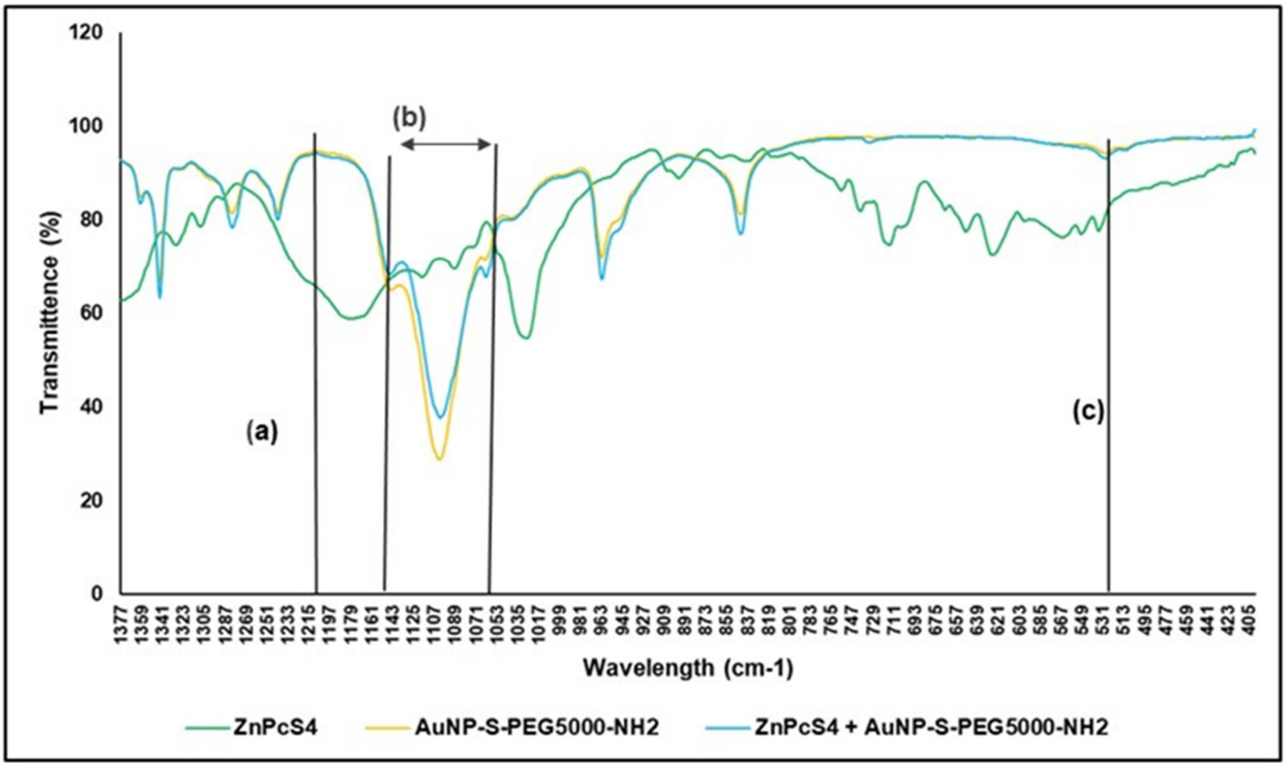

Figure S3: FT-IR spectra analysis for confirmatory ligand exchange (Au-S) and absorption (S-S) bond confirmation between AuNP-S-PEG5000-NH_2 and $ZnPcS_4$ PS within the final BNC. (a) C-S (1200 cm-1 Sharp): Au-S ligand exchange bond formed as $ZnPcS_4$ PS lost its C-S groups to form an Au-S bond with the AuNPs, (b) C-S (1050 – 1200 cm-1 Stretch): Au-S ligand exchange bond formed as AuNPs PS lost its C-S groups to form an Au-S bond with $ZnPcS_4$ PS and (c) S-S (500 – 540 cm-1 Bend): Disulphide (SS) bond formed due to ligand exchange process being performed in air causing $ZnPcS_4$ to form a dimer.

Moreover, Au-S ligand exchange bond formation was confirmed since the FTIR spectra of AuNP-S-PEG5000-NH_2 + $ZnPcS_4$ noted a loss of a C-S (1200 cm^{-1}) sharp band when compared to $ZnPcS_4$ PS alone, suggesting $ZnPcS_4$ PS lost its C-S groups to bond with AuNP-S-PEG5000-NH_2 (Figure S3) [8]. Finally, the FTIR spectra of AuNP-S-PEG5000-NH_2 + $ZnPcS_4$ PS exhibited a sharp S-S (500-540 cm^{-1}) band indicating weak di-sulphide bond formed due to the ligand exchange process being performed in air and so the $ZnPcS_4$ PS formed a dimer (Figure S3) [8–10].

Additionally, the final BNC underwent FTI-R analysis for confirmatory amide bond analysis, by examining and identifying the formation of amide bonds when compared to the FTIR spectra of AuNP-S-PEG5000-NH_2 alone (Figure S4). With reference to Figure S4, the presence of the C=O band within the final BNC confirms amide bond formation alone, but the N=H band is not clearly evident, when compared to the AuNP-S-PEG5000-NH_2 alone, signifying that strong primary and secondary amide bonds (CO-NH) had formed between the amine (NH) functionalized group on the AuNPs and the activated c' terminus of the Anti-GCC Ab [2], suggesting that thee BNC was capable of active targeting.

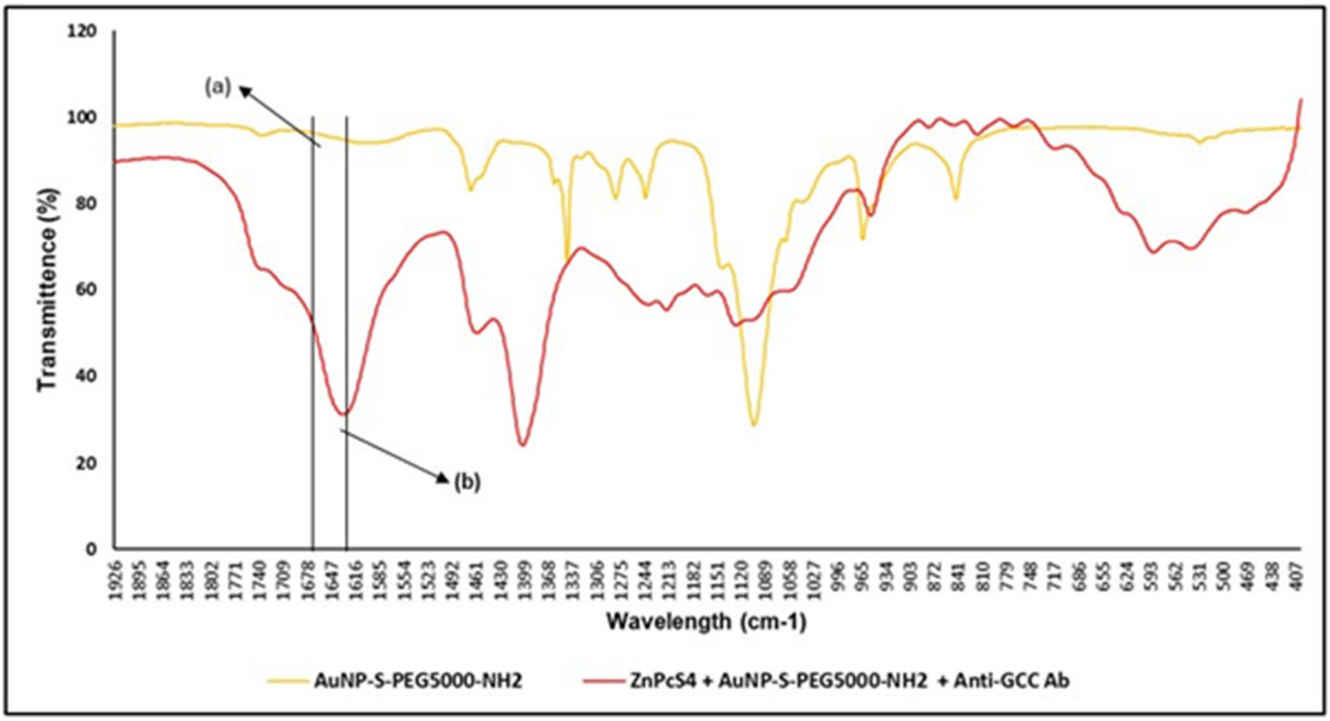

Figure S4: FT-IR spectra analysis for confirmatory amide (CO-NH) bond confirmation between AuNP-S-PEG5000-NH_2 and the activated carboxylic group on the c' terminus of the Anti-GCC Ab within the final BNC. (a) C=O (1680 – 1630cm^{-1} Stretch): Amide peptide bond (CO-NH) formed between amine (NH) AuNP functional group and activated carboxylic group on the c' terminus of the Ab and (b) N-H (1640 – 1650 cm^{-1} Bend): Secondary amide (NH) bond formed between amine (NH) AuNP functional group and activated carboxylic group on the c' terminus of the Ab.

2.2.3 Dynamic Light Scattering (DLS) and Zeta Potential (ZP)

DLS and ZP results are indicated in Table S2 and Figure S5. The final BNC produced one single major peak (repeated in triplicate) with a narrow width and no additional smaller side peaks, suggesting that it was homogenously pure, remained spherical in shape and reported no aggregation (Figure S5) [11]. The BNC noted a mean desirable Z-average of 57.18 ± 3.04 nm.

With reference to Table S2, it can be noted that the final BNC noted a PDI value of 0.353, signifying it was monodispersed in nature and consisted mostly of singular sized particles [11]. Moreover, these results imply

that the three individual constituents ($ZnPcS_4$, AuNP-S-PEG5000-NH_2 and Anti-GCC mAb) were successfully bonded together to form one single final BNC. The ZP value of the final BNC was 36.5 ± 2.6 mV, suggesting that it is highly stable with a slightly positively charge and so should remain stable under *in vivo* conditions, as well as be retained within cancer tumour cells far more selectively [11,12].

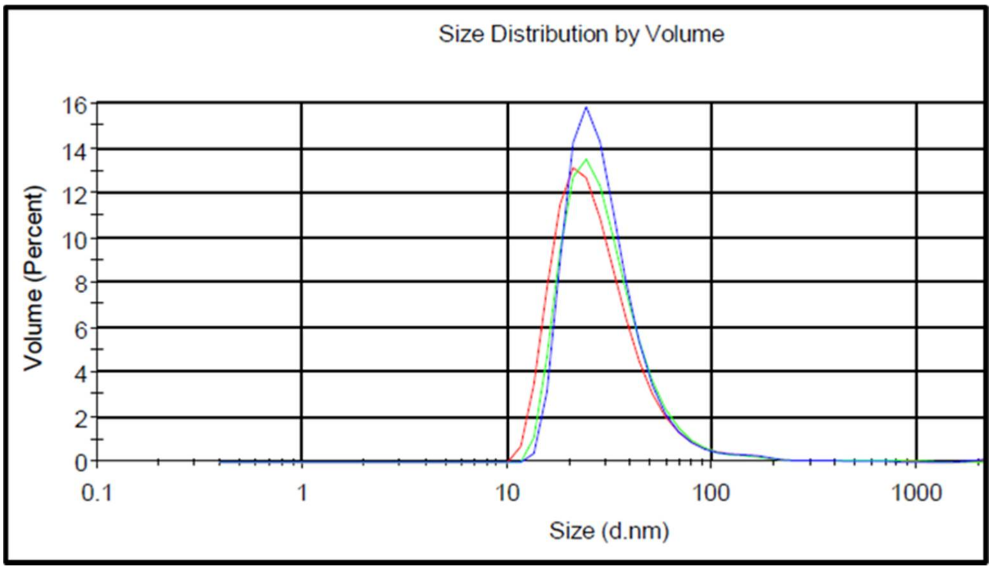

Figure S5: DLS hydrodynamic radius distribution graph of the final BNC.

Table S2: DLS and ZP characterization results for the final BNC

Sample	Mean Z-Average Diameter Measured by DLS (nm)	Polydispersity Index (PDI)	Zeta Potential (mV)
AuNP	11.78 ± 0.966	0.351 (Monodisperse)	
$ZnPcS_4$	18.15 ± 1.44	0.107(Monodisperse)	
Expected average $ZnPcS_4$ - AuNP	48.08 ± 1.20		
Result average $ZnPcS_4$ - AuNP	44.57 ± 0.564	0.250 (Monodisperse)	
Anti-GCC Ab	5.21 ± 0.51	0.400 (Monodisperse)	
Expected average $ZnPcS_4$ - AuNP - Anti-GCC Ab	54.99 ± 0.537		
Result average $ZnPcS_4$ - AuNP - Anti-GCC Ab (final BNC)	57.18 ± 3.04	0.353 (Monodisperse)	36.5 ± 2.6 (Highly stable)

REFERENCE

1. Naidoo, C.; Kruger, C.A.; Abrahamse, H. Targeted Photodynamic Therapy Treatment of in Vitro A375 Metastatic Melanoma Cells. *Oncotarget* **2019**, *10*, 6079–6095, doi:10.18632/oncotarget.27221.

2. Jazayeri, M.H.; Amani, H.; Pourfatollah, A.A.; Pazoki-Toroudi, H.; Sedighimoghaddam, B. Various Methods of Gold Nanoparticles (GNPs) Conjugation to Antibodies. *Sensing and Bio-Sensing Research* **2016**, *9*, 17–22, doi:10.1016/j.sbsr.2016.04.002.

3. Avelar-Freitas, B.A.; Almeida, V.G.; Pinto, M.C.X.; Mourão, F. a. G.; Massensini, A.R.; Martins-Filho, O.A.; Rocha-Vieira, E.; Brito-Melo, G.E.A. Trypan Blue Exclusion Assay by Flow Cytometry. *Braz J Med Biol Res* **2014**, *47*, 307–315, doi:10.1590/1414-431X20143437.

4. Manoto, S.; Sekhejane, P.; Houreld, N.; Abrahamse, H. Localization and Phototoxic Effect of Zinc Sulfophthalocyanine Photosensitizer in Human Colon (DLD-1) and Lung (A549) Carcinoma Cells (in Vitro). *Photodiagnosis and photodynamic therapy* **2012**, doi:10.1016/j.pdpdt.2011.08.006.

5. Ekka, D.; Roy, M.N. Quantitative and Qualitative Analysis of Ionic Solvation of Individual Ions of Imidazolium Based Ionic Liquids in Significant Solution Systems by Conductance and FT-IR Spectroscopy. *RSC Adv.* **2014**, *4*, 19831–19845, doi:10.1039/C3RA48051H.

6. Nguyen, K.C. Quantitative Analysis of COOH-Terminated Alkanethiol SAMs on Gold Nanoparticle Surfaces. *Advances in Natural Sciences: Nanoscience and Nanotechnology* **2012**, *3*, 045008, doi:10.1088/2043-6262/3/4/045008.

7. Mecozzi, M.; Sturchio, E. Computer Assisted Examination of Infrared and Near Infrared Spectra to Assess Structural and Molecular Changes in Biological Samples Exposed to Pollutants: A Case of Study. *J. Imaging* **2017**, doi:10.3390/jimaging3010011.

8. Siriwardana, K.; Gadogbe, M.; Ansar, S.; Vasquez, E.; Collier, W.; Zou, S.; Walters, K.; Zhang, D. Ligand Adsorption and Exchange on Pegylated Gold Nanoparticles. *The Journal of Physical Chemistry C* **2014**, *118*, doi:10.1021/jp501391x.

9. Nombona, N.; Antunes, E.; Litwinski, C.; Nyokong, T. Synthesis and Photophysical Studies of Phthalocyanine–Gold Nanoparticle Conjugates. *Dalton Trans.* **2011**, *40*, 11876–11884, doi:10.1039/C1DT11151E.

10. Chechik, V. Reduced Reactivity of Aged Au Nanoparticles in Ligand Exchange Reactions. *J. Am. Chem. Soc.* **2004**, *126*, 7780–7781, doi:10.1021/ja048879w.

11. Bhattacharjee, S. DLS and Zeta Potential - What They Are and What They Are Not? *J Control Release* **2016**, *235*, 337–351, doi:10.1016/j.jconrel.2016.06.017.

12. Patel, V.; Agrawal, Y. Nanosuspension: An Approach to Enhance Solubility of Drugs. *Journal of advanced pharmaceutical technology & research* **2011**, *2*, 81–7, doi:10.4103/2231-4040.82950.
